# Bayesian inferences suggest that Amazon Yunga Natives diverged from Andeans less than 5000 ybp: implications for South American prehistory

**DOI:** 10.1186/s12862-014-0174-3

**Published:** 2014-09-30

**Authors:** Marilia O Scliar, Mateus H Gouveia, Andrea Benazzo, Silvia Ghirotto, Nelson JR Fagundes, Thiago P Leal, Wagner CS Magalhães, Latife Pereira, Maira R Rodrigues, Giordano B Soares-Souza, Lilia Cabrera, Douglas E Berg, Robert H Gilman, Giorgio Bertorelle, Eduardo Tarazona-Santos

**Affiliations:** Departamento de Biologia Geral, Instituto de Ciências Biológicas, Universidade Federal de Minas Gerais, Belo Horizonte, Brazil; Dipartimento di Scienze della Vita e Biotecnologie, Università di Ferrara, Ferrara, Italy; Departamento de Genética, Instituto de Biociências, Universidade Federal do Rio Grande do Sul, Porto Alegre, Brazil; Asociación Benéfica PRISMA, Lima, Peru; Department of Molecular Microbiology, Washington University School of Medicine, St. Louis, USA; Department of Medicine, University of California San Diego, San Diego, USA; Bloomberg School of Public Health, Johns Hopkins University, Baltimore, USA; Universidad Peruana Cayetano Heredia, Lima, Peru

**Keywords:** Population genetics inferences, Human evolution, Native American

## Abstract

**Background:**

Archaeology reports millenary cultural contacts between Peruvian Coast-Andes and the Amazon Yunga, a rainforest transitional region between Andes and Lower Amazonia. To clarify the relationships between cultural and biological evolution of these populations, in particular between Amazon Yungas and Andeans, we used DNA-sequence data, a model-based Bayesian approach and several statistical validations to infer a set of demographic parameters.

**Results:**

We found that the genetic diversity of the Shimaa (an Amazon Yunga population) is a subset of that of Quechuas from Central-Andes. Using the Isolation-with-Migration population genetics model, we inferred that the Shimaa ancestors were a small subgroup that split less than 5300 years ago (after the development of complex societies) from an ancestral Andean population. After the split, the most plausible scenario compatible with our results is that the ancestors of Shimaas moved toward the Peruvian Amazon Yunga and incorporated the culture and language of some of their neighbors, but not a substantial amount of their genes. We validated our results using Approximate Bayesian Computations, posterior predictive tests and the analysis of pseudo-observed datasets.

**Conclusions:**

We presented a case study in which model-based Bayesian approaches, combined with necessary statistical validations, shed light into the prehistoric demographic relationship between Andeans and a population from the Amazon Yunga. Our results offer a testable model for the peopling of this large transitional environmental region between the Andes and the Lower Amazonia. However, studies on larger samples and involving more populations of these regions are necessary to confirm if the predominant Andean biological origin of the Shimaas is the rule, and not the exception.

**Electronic supplementary material:**

The online version of this article (doi:10.1186/s12862-014-0174-3) contains supplementary material, which is available to authorized users.

## Background

Knowing how Native Americans dispersed along the American continent is still a major challenge faced by researchers studying the biological and cultural evolution of the region [1-3]. Also, how natives adapted to diverse environmental challenges such as hypoxia and cold weather in the Andes [4] and the tropical forest [5] remain poorly understood.

When Europeans arrived in South America in the 16th century, the Pan-Andean Inca Empire dominated the Andean region and had a population density and levels of socioeconomic development unmatched elsewhere in South America. But the Inca Empire is just the tip of the iceberg of a long-term cultural and biological evolutionary process that involved the entire Andean region and its adjacent Pacific Coast (hereafter western South America). This process began 14–11 thousand years BP, with the peopling of this region in the Late Pleistocene [2], involving continuous cultural exchanges and gene flow along time, and leading to a relative genetic, cultural, and linguistic homogeneity between the populations of western South America when compared with eastern South America (a term that hereafter refers to the eastern region of the Andes, including the low Amazon Basin), where populations remained relatively more isolated [6] than those in western South America. For example, only two languages still predominate across the entire Andean region (Quechua and Aymara), whereas in eastern South America natives speak a wider spectrum of languages belonging to four different linguistic families [7]. Also, despite some controversy about definitions and chronology, archeologists consensually recognize three temporal Horizons in the Andes and the Pacific Coast (Early, Middle, and Late). Each of the Horizons corresponds to periods of material cultural dispersion involving a wide geographic area and, in the case of Middle and Late Horizons, to the expansion of the Wari-Tiwanaku and Inca States, respectively [8].

The current knowledge about western South American prehistory derives mainly from a plethora of archeological studies [9], most of which have focused on the Pacific Coast and Andean people. However, the relationships between Andeans and their culturally, linguistically, and environmentally different eastern neighbors living in the Amazon Yunga remain relatively neglected by archeologists, despite early investigations by Lathrap [10] and some subsequent studies that have been done on the subject [11]. Notwithstanding this knowledge gap, the Amazon Yunga, a region hosting at least six ethnic groups, is particularly interesting because it is a transitional environment between the Andes highlands and the lowland tropical forest of the Amazonia. Moreover, archeological research in the lowland Amazonia during the last decades has changed the traditional view of the Amazonian environment as incompatible with complex pre-Columbian societies [12]. The emerging view, that has gained growing support, recognizes that the Amazonian basin has hosted the earliest ceramics of South America, that endogenous agricultural societies with complex organization have developed there, and that population sizes were larger than previously thought [13,14]. In contrast, information derived from anthropological genetics is scantier, especially for Amazonian and Amazon Yunga populations [15-18]. Contributing to our poor understating of how these populations evolved is the fact that Native Americans are under-represented in modern genetic studies [19] due to cultural issues and logistic difficulties in reaching them in the tropical forest.

Cultural and commercial interactions occurring along the last millennia among the people living in the Peruvian Coast, the Andes and the Amazon Yunga regions are archaeologically documented. For example, cultivated plants such as sweet potato and manioc, ceramic iconography and styles (Tutishcanyo, Kotosh, and Valdivia) and traditional coca chewing [14] have been shared among Coast, Andean and Amazon Yunga populations. However, we ignore how the demographic evolutionary history of these populations accompanied their cultural and socioeconomic interactions. Specifically, and this is the goal of this study, we aimed to investigate the demographic relationships between the Shimaa population and their western Andean neighbors. Our study is the first to analyze a sample of the Amazon Yunga population at a multilocus level. Here we show that the genetic diversity of the Shimaa, a Matsiguenga Arawak-speaking population settled in and with an Amazon Yunga lifestyle, is a subset of the Andean Quechua diversity. We used a Bayesian inference framework and several statistical validation tools to infer that the Shimaa likely originated from an ancestral Andean population less than 5300 years ago, around or after the time when complex societies in the Andean region emerged.

## Results and discussion

**Figure 1:**
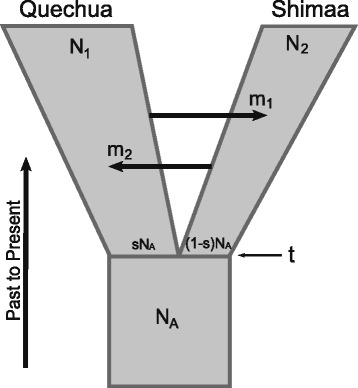
**Isolation with Migration (IM) model.** The IM model includes an ancestral population of effective size of **N**_**A**_ individuals that split **t** generations ago in two populations, one of size **sN**_**A**_ and the other of size **(1-s)N**_**A**,_ where s ∈ [0,1]. Their sizes are allowed to change exponentially to their current effective sizes **N**_**1**_ (Quechua) and **N**_**2**_ (Shimaa)_**.**_ Over **t** generations, gene flow can occur between the two descendant populations at different rates in both directions **(m**_**1**_ **and m**_**2**_ **)**.

We used genetic data and a population genetic model to infer the evolutionary relationships between a Quechua population from the Peruvian Central Andean Highlands and an Arawak Matsiguenga population (Shimaa) from the Southern Peruvian Amazon Yunga (Additional file 1: Figure S1). These populations, separated by 300 km, speak languages from different families (Pan-Andean Quechua and Arawak Matsiguenga, respectively) and have different cultures related to their high altitude Andean and rainforest Amazon Yunga lifestyles, respectively. We sequenced 10 independent genomic regions for a total of ~20 kb per individual [20] in 11 Quechua and 10 Shimaa individuals, for whom we estimated [21] negligible non-native genetic contribution (<5% in Quechuas in Scliar et al. [22], and ~1% in Shimaas, Additional file 1: Figure S2), based on genotyping of 106 Ancestry Informative Markers [23]. We used a model-based Bayesian approach to infer the posterior distributions of a set of demographic parameters [24]. The tested model considers and distinguishes the effects of genetic drift after the split of two populations and the subsequent gene flow between them in an explicit probabilistic framework (Figure 1). We inferred the parameters of the model using the likelihood-based method implemented in the software IM. This method uses the entire information provided by the data and applies a Markov Chain Monte Carlo (MCMC) computational approach [24]. Even though this model reduces the complex history of two populations, that actually evolve together with surrounding groups, to an only-two-populations system, simulation studies have shown that inferences are robust, despite moderate violations of the model assumptions, which were simulated to mimic comparable situations often encountered in real-world scenarios [25].

**Figure 2:**
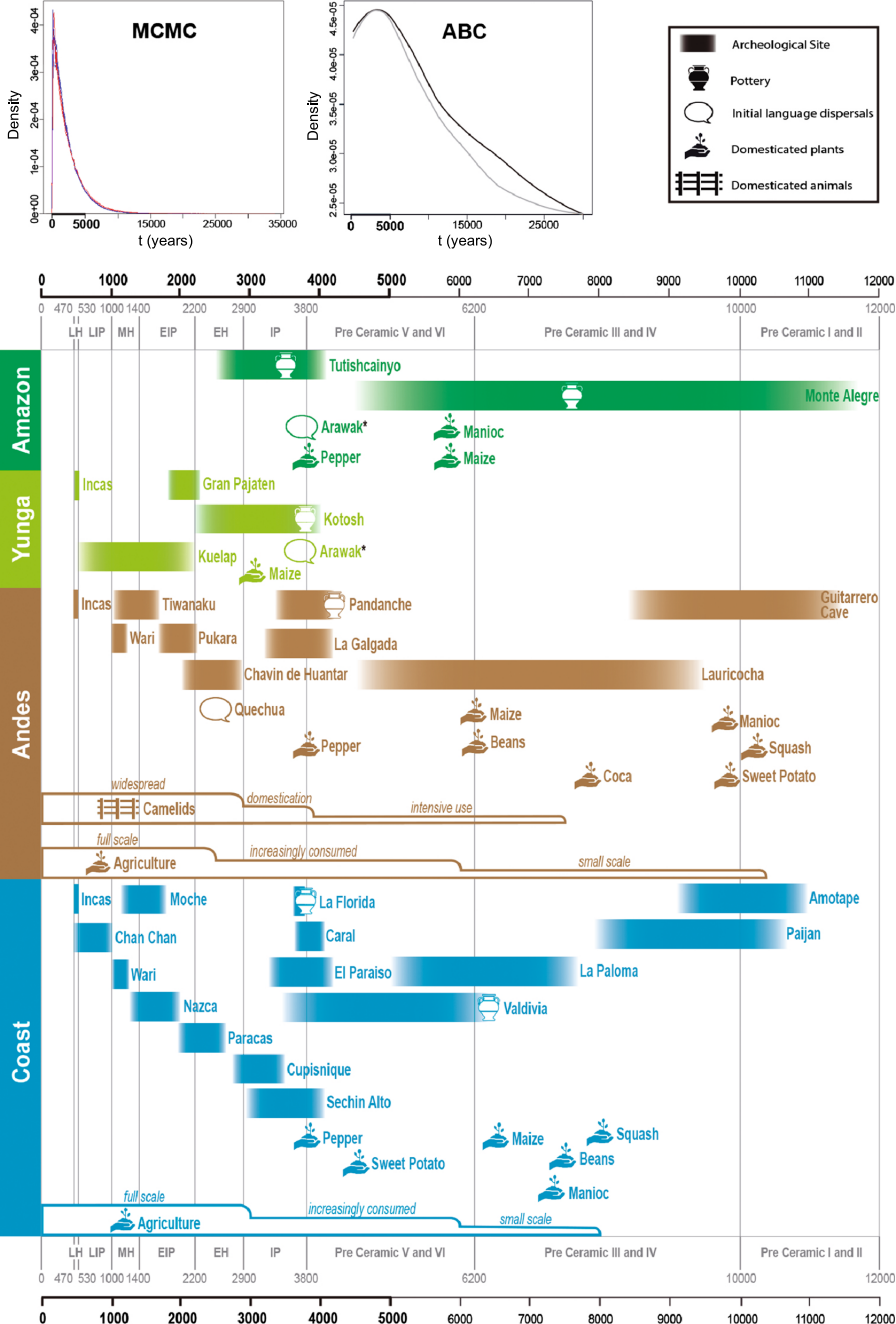
**Posterior probabilities for the time of divergence between Quechua and Shimaa in its historical context.** Posterior probability densities for the time of divergence t (years) between Quechua and Shimaa populations, obtained by MCMC and ABC, in its historical context. The period encompassing the 90% HPD (Highest Posterior Density) interval of the posterior probability of **t**, estimated by MCMC is highlighted. **MCMC plot**: Red: three independent runs with migration rate parameters M_i_ = 10; Blue: three independent runs with migration rate parameters M_i_ = 0. **ABC plot**: Gray: model without intra-locus recombination; Black: model with intra-locus recombination. Below are key historical events of Peruvian prehistory in four Peruvian longitudinal regions: Coast, Andes, Amazon Yunga and Amazonia. Pottery and cultivars symbols represent the earliest archaeological record for the region. This chronology is a simplified picture of the Peruvian archaeological history in which we used different dating records for its construction. To account for time uncertainties, we depicted the events in the chronology plot without clearly defined chronological borders. References for the historical events presented are specified in Additional file 2. LH: Late Horizon, LIP: Late Intermediate Period, MH: Middle Horizon, EIP: Early Intermediate Period, EH: Early Horizon, IP: Initial Period. *Controversial geographic region of Arawak origin. Each step in Agriculture and Camelids representations shows an increase in their relative importance.

**Figure 3:**
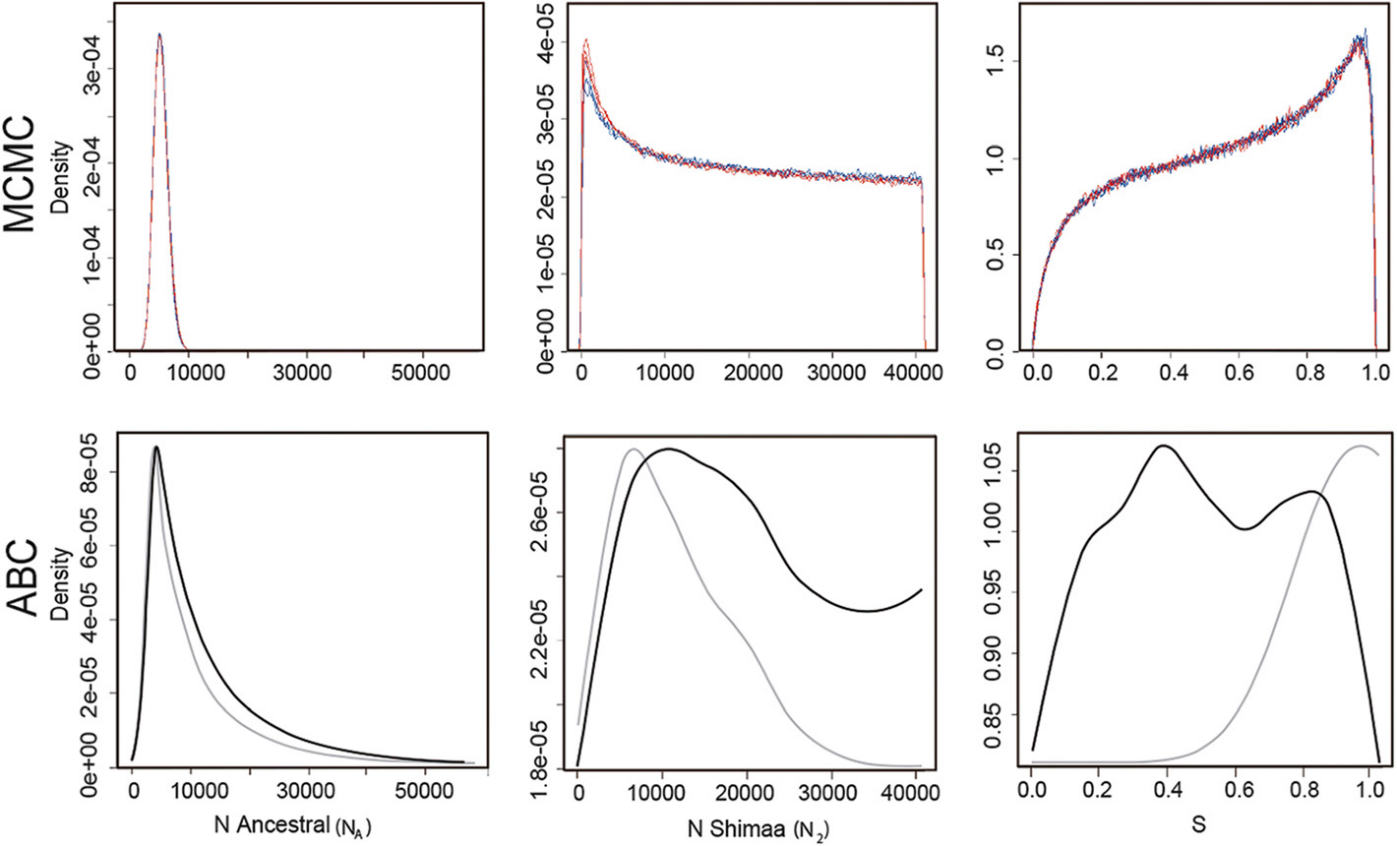
**Posterior probabilities for the parameters N**_**A**_ **, N**_**2**_ **, and s.** Posterior probability densities obtained by the MCMC method and by the ABC for the parameters N for ancestral effective size (N_A_), N Shimaa effective size (N_2_), and the s parameter (the proportion of N_A_ that founded the Quechua (N_1_) population. Range of prior probability distributions are in Additional file 1: Table S2. **N**_**i**_ are in number of individuals. **MCMC plots**: Red: three independent runs with migration rate parameters M_i_ = 10; Blue: three independent runs with migration rate parameters M_i_ = 0. **ABC plots**: Gray: model with no intra-locus recombination; Black: model with intra-locus recombination. Posterior probabilities for N Quechua **(N**_**1**_ **)**, **m**_**1**_ and **m**_**2**_ did not yield informative densities and are presented in Additional file 1: Figure S3.

We found that Amazon Yunga Shimaas have a low genetic diversity that interestingly, is a subset of the higher diversity observed in the Andean Quechuas. In fact, more SNPs are found in the Quechuas than in the Shimaas and all the SNPs found in the Shimaa are shared with the Quechuas (Additional file 1: Table S3). The distribution of the mass probability of the divergence time posterior density (Figure 2 and Table 1) suggests that the Quechua and the Shimaa populations diverged recently: If the point estimate appears implausible, the upper limit of the 90% density interval excludes a divergence older than 5300 years ago. At the time of the split, individuals carrying s ≈ 96% (Figure 3, Table 1) of the effective population size of the ancestral population founded the Quechua population, while only a small fraction (s ≈ 4%) founded the Shimaa. This statement does not necessarily mean that the individuals that founded the Shimaa were around 1/25 of the ancestral population. Instead, in population genetics, the definition of effective population size implies that respect to the ancestral Quechua population, the ancestral Shimaa population behaved as an ideal Wright-Fisher model population that lost diversity due to the action of the genetic drift at a pace around twenty-five times faster. Therefore, the lower effective population of the Shimaa may have resulted from a combination of a certainly much smaller number of individuals together with other factors known to reduce the effective population size, such as a biased sex ratio or a high variance in the number of progeny [26].

**Table 1:** **Estimates of demographic parameters**

**Demographic parameters**	**MCMC**	**ABC**	**ABC_rec**
Time split (t)	193 (15–5291)	3300 (250–26010)	3377 (250–25956)
N Ancestral (N_A_)	5475 (3766–7702)	3829 (741–25863)	4220 (821–29290)
N Shimaa (N_2_)	681^a^	6449 (5–34640)	10641^a^
s^b^	0.96 (0.17–0.99)	0.89 (0.21–0.99)	-^c^

Population size in number of individuals and time split in years. The estimates are the mode of the posterior distribution with the 90% HPD (Highest Posterior Density) interval between parentheses. MCMC estimates are the averages over six runs.

^a^We did not specified the 90% HPD for these estimates, because the right end of the posterior distribution did not approach zero in the vertical axis before the upper boundary of the prior distribution. This implies that the 90% HPD depends on the definition of the prior distribution.

^b^Fraction of the ancestral population that founded the Quechua population.

^c^This parameter did not yield an informative density.

We validated our MCMC results by assessing the statistical convergence of multiple MCMC runs and by performing posterior predictive tests [27], and both procedures confirmed our inferences (Additional file 1: Section 2.1). To further validate our results, and because the IM model implemented in the MCMC framework [24] does not consider genetic intra-locus recombination, we also inferred the model parameters (Figure 1) by Approximate Bayesian Computations (ABC) [28], although our analyzed dataset shows almost no intra-locus recombination (see Methods). While the MCMC-approach uses the complete dataset at a cost of less flexibility of the model (for example, not allowing recombination), ABC allows more flexibility (in this case, the inclusion of recombination), but it only uses summary statistics to compare simulated and real data. This suboptimal use by ABC of the available information in analyzed datasets comes at a cost of larger credible intervals [29]. To analyze the consequences of considering recombination in the ABC, we did one analysis with recombination (ABC_rec) and one without recombination (ABC). The ABC estimates of the ancestral population size and the relative ratio of the two population sizes at the time of the split are similar to the IM estimates (Table 1). We inferred an earlier population divergence, though the point estimates with ABC is still within the 90% interval obtained with the MCMC approach. We found, however, that ABC tended to overestimate the divergence time with our data (see pseudo-observed datasets validation in Additional file 1: Section 2.2). Similar results are obtained regardless the inclusion of recombination in the ABC. Overall, our ABC estimates confirm our MCMC results (Figures 2 and 3, Table 1, and Additional file 1: Section 2.2).

Our results indicate that the Shimaa diverged from the Quechua Andean population after the late Pleistocene peopling of South America, and very likely, less than 5300 years ago. The 5000–3000 years period BP was characterized by the development of complex societies in the Andean Region and the Pacific Coast, being a period of major cultural development, when large permanent communities settled, monumental architecture appeared, pottery came into use, and agriculture became the predominant source of food supply (see Figure 2 and its references in Additional file 2). Representative settlements of that time were Kotosh in the Amazon Yunga (Huanuco Region), La Galgada in the Central Andes, and Caral and El Paraiso in the Central Pacific Coast (Figure 2). The initial dispersal of the Arawak, which is the language currently spoken by the Shimaa, also seems to have occurred during this time [29], and some authors (see Figure 2), suggest this linguistic family has originated in the Peruvian Amazon Yunga [30-32]. The most plausible scenario compatible with our results is that a small subpopulation (the ancestors of the Shimaa) split from a larger Andean population, moved toward the Peruvian Amazon Yunga and then adapted to the different lifestyle of the Amazon Yunga, incorporating the culture of some of their neighbors and their language (Arawak), but not a substantial amount of their genes.

## Conclusions

The question about the evolutionary relationship between Andean and the Lower Amazonian populations (i.e. if they derived from different migration routes into South America) is still open in American anthropology. We contributed to clarify -for the first time using multilocus data; the prehistoric demographic relationship between Andeans and a population from the Amazon Yunga. Moreover, our results offer a testable model for the peopling of this large transitional environmental region between the Andes and the Lower Amazonia. Andean populations are highly homogeneous [6,15-18] which supports our assumption that the Quechua sample used in this study is a fair representative of Andean populations. Nevertheless, further studies on the populations of the Amazon Yunga and the Lower Amazonia are necessary to show if the predominant Andean biological origin of the Shimaas and its pattern of adaptation to the new environment is the rule, and not the exception.

## Methods

### Dataset

We studied Native Americans from two Peruvian populations: (i) 11 Andean Quechua individuals reported in Scliar et al. [22] and (ii) 10 Matsiguenga individuals from the Shimaa population, randomly selected from a total sample of 180 individuals available at our laboratory. The Matsiguenga are settled in this area of the Amazon Yunga since the 16th century [33]. This study was conducted under approval of the Institutional Reviews Boards from the Universidad Peruana Cayetano Heredia, Asociación Benéfica PRISMA, Universidade Federal de Minas Gerais and Johns Hopkins University. Detailed information about the re-sequencing of the 10 autosomal non-coding unlinked loci [20] used in this study is available in Scliar et al. [22] and in Additional file 1: Section 1.1. Sequence analysis were performed following the pipeline specified in Machado et al. [34]. We used 106 Ancestry Informative Markers [23] to perform admixture analyses of these individuals, using the software Structure [21], as detailed in Additional file 1: Section 1.2. The Shimaa dataset is available in GenBank [GenBank: KF690381-KF690580]. Additional file 3 presents the individual genotypes of the dataset used for the analysis that contains the 52 segregating sites identified in this study.

### MCMC inferences

We used the IM program, that uses MCMC simulations of genealogies to estimate seven parameters of the Isolation-with-Migration model depicted in Figure 1 [24,35]: three population mutation rate parameters for the ancestral and the two descendant populations (θ_A_, θ_1_, and θ_2_, respectively, where θ = 4 N_e_μ); the splitting time parameter (T = tμ); the ratio of migration rate per mutation rate, in both directions (M_1_ = m_1_/μ and M_2_ = m_2_/μ), and the proportion of the ancestral population that founded population 1 (s) [24]. We assumed the infinite-site mutation model [36] for all loci. Except for s, the other model parameters are scaled by the neutral mutation rate μ. Therefore, to obtain the demographic estimates N_e_, t and m, a mutation rate needs to be assumed. Mutation rates for each locus were estimated using the BEAST software [37] assuming a divergence time of 6 million years between humans and chimpanzee. Under the multilocus model, the mutation rate is the geometric mean of the individual locus-specific mutation rates [24]. We used the geometric mean per year (1.47 × 10^−6^) to obtain the estimated time since splitting, t, in years and migration rate, m, per year. To obtain N_e_, a measure of mutation rate on a scale of generations is needed. We assumed 25 years/generation, which yield a geometric mean value of 3.68 × 10^−5^ mutations per generation.

Because the IM model originally assumes no recombination within loci, we used the program IMgc to find the largest subset of the data containing no signs of recombination. IMgc uses the four-gametes criteria to remove either sequences or variable sites containing evidence of recombination [38]. This procedure resulted in the removal of sequences QT80 and QT135 for locus 4, of sequence QA38 for locus 5, and of the second half segment of locus 3.

Three independent MCMC runs were performed, each with 30 Metropolis-coupled chains of 10 million steps using a geometric heating scheme and a burn-in period. Prior uniform distributions were defined as follows: θ_i_ ∈ (0, 5), T∈ (0, 0.05), M_i_ ∈ (0, 10), and s ∈ (0, 1). These scaled values correspond to the values specified in Additional file 1: Table S2. We also performed three independent runs without migration (M_i_ = 0 as prior), with 30 Metropolis-coupled chains of 8 million steps. We compared the results from the runs with and without migration, because Kitchen et al. [39] identified important changes in the estimates when using different migration rates as priors. Additional file 1: Section 1.3 details the criteria used to check convergence and MCMC results validation by posterior predictive test [27,40-42].

### Inferences by ABC

To validate the results of the MCMC method by incorporating genetic intra-locus recombination (not allowed in the MCMC-IM model) in our analyses, we used ABC [28,43] as a more general model framework, to infer the same seven demographic parameters of the MCMC model. We performed one analysis with recombination (ABC_rec) and one without recombination (ABC). The ABC approach approximates the posterior distribution by performing a large number of simulations under a specific model and calculating the distance between Summary Statistics (SuSt) estimated from the simulated data and SuSt estimated from the observed data. We used the program fastsimcoal within the ABCToolBox for simulations [44-46]. Used prior distributions are given in Additional file 1: Table S2 and its rationale explained in the Additional file 1: Section 1.4 when necessary. SuSt used in the ABC analyses are explained in detail in the Additional file 1: Section 1.4. Additional file 1: Table S3 presents the SuSt estimates for the observed data. For parameter estimation, we calculated the Euclidian distance between the simulated and observed SuSt and retained the 1% of the total simulations corresponding to the shortest distances. Posterior probability for each parameter was estimated using a weighted local regression [28]. We assessed the quality of the parameters estimated by ABC by assessing the determination coefficient R^2^ (the proportion of parameter variance explained by the summary statistics), by analyzing pseudo-observed datasets and by posterior predictive tests [27], as detailed in the Additional file 1: Section 1.5.

## Availability of supporting data section

New sequence data generated for this study have been deposited in GenBank (http://www.ncbi.nlm.nih.gov/genbank) under accession numbers KF690381 to KF690580.

## Additional files

Additional file 1: **Contain supplementary material: the file includes supplementary methods description, supplementary results, Tables S1-S7, Figures S1-S4, and references exclusive of Additional file 1.**

Additional file 2: **References used to prepare Figure** **2** **.**

Additional file 3: **Individual genotypes of the dataset used for the analysis, that contains the 52 segregating sites identified in this study.**
