# Supplementary material for: Bayesian inferences suggest that Amazon Yunga Natives diverged from Andeans less than 5000 ybp: implications for South American prehistory
Source: BMC Evol Biol. 2014 Sep 30;14:174. doi: 10.1186/s12862-014-0174-3 (PMC4189748; doi:10.1186/s12862-014-0174-3)
Supplement: Supplementary file 1 — Contain supplementary material: the file includes supplementary methods description, supplementary results, Tables S1-S7, Figures S1-S4, and references exclusive of Additional file 1. [file 12862_2014_174_MOESM1_ESM.docx]

**Additional file 1**

**Title:** BAYESIAN INFERENCES SUGGEST THAT AMAZON YUNGA NATIVES DIVERGED FROM ANDEANS LESS THAN 5000 YBP: IMPLICATIONS FOR SOUTH AMERICAN PREHISTORY

**Marilia O Scliar^1^, Mateus H Gouveia^1^, Andrea Benazzo^2^, Silvia Ghirotto^2^, Nelson J Fagundes^3^, Thiago P Leal^1^, Wagner CS Magalhães^1^, Latife Pereira^1^, Maira R Rodrigues^1^, Giordano Soares-Souza^1^, Lilia Cabrera^4^, Douglas E Berg^5,6^, Robert H Gilman^4,7,8^, Giorgio Bertorelle^2^, Eduardo Tarazona-Santos^1§^**

^1^ Departamento de Biologia Geral, Instituto de Ciências Biológicas, Universidade Federal de Minas Gerais, Belo Horizonte, Brazil

^2^ Dipartimento di Scienze della Vita e Biotecnologie, Università di Ferrara, Ferrara, Italy

^3^ Departamento de Genética, Instituto de Biociências, Universidade Federal do Rio Grande do Sul, Porto Alegre, Brazil

^4^ Asociación Benéfica PRISMA, Lima, Peru

^5^ Department of Molecular Microbiology, Washington University School of Medicine, St. Louis, USA

^6^ Department of Medicine, University of California San Diego, San Diego, USA

^7^ Bloomberg School of Public Health, Johns Hopkins University, Baltimore, USA

^8^ Universidad Peruana Cayetano Heredia, Lima, Peru

**INDEX**

[**1.** **MATERIALS AND METHODS** 3](#_Toc389159742)

[**1.1.** **Re-sequencing dataset** 3](#_Toc389159743)

[**1.2.** **Admixture analyses** 4](#_Toc389159744)

[**1.3. Validation of the MCMC results** 4](#_Toc389159745)

[**1.4.** **Inferences using Approximate Bayesian Computation (ABC)** 5](#_Toc389159746)

[**1.5.** **Validation of ABC results** 6](#_Toc389159747)

[**2.** **RESULTS** 7](#_Toc389159748)

[**2.1. Validation of MCMC results** 7](#_Toc389159749)

[**2.2. Validation of ABC results and comparison between ABC and ABC_rec** 8](#_Toc389159750)

[**3.** **SUPPLEMENTARY TABLES** 9](#_Toc389159751)

[**4.** **SUPPLEMENTARY FIGURES** 19](#_Toc389159752)

[**5.** **REFERENCES** 25](#_Toc389159753)

# **MATERIALS AND METHODS**

## **Re-sequencing dataset**

We re-sequenced Native Americans from two Peruvian populations: (i) 10 Quechua individuals from the farmer community of Tayacaja (12°20’S, 75°50'W), Province of Huancavelica, and one Quechua individual from Arequipa, both from Peruvian Central Andes, previously reported in Scliar et al. (*22*) (total of 11 Quechua individuals); (ii) 10 Matsiguenga individuals from the Shimaa population (12°33'S, 73°05'W), which is one of the 34 Matsiguenga communities settled in the Peruvian Amazon Yunga. The Shimaa encompasses around 85 families living in small villages (www.selvasperu.org). The Matsiguenga are one of six Arawak peoples that currently live in the Peruvian Amazon Yunga. The historical records reveal that the Matsiguenga already inhabited this same area since the 16th century (*33*). Samples were collected after individuals have signed the informed consent and study was conducted under approval from the Institutional Reviews Boards from the Universidad Peruana Cayetano Heredia, Asociación Benéfica PRISMA and Universidade Federal de Minas Gerais.

We bi-directionally re-sequenced 10 autosomal non-coding unlinked loci described by Frisse et al. (*20*) from nine different autosomes. Each sequenced region spans a locus-pair, i.e. two segments of 1-2 kb at each end of a ~10-kb segment. We re-sequenced a total of 20 kb from each individual, including the 10 locus-pairs, except for region 6, which was re-sequenced only for the second segment. Detailed methodological information about the re-sequencing scheme is available in Scliar et al. (*22*). The Shimaa dataset has been submitted to GenBank under accession numbers KF690381-KF690580.

Although only 1.7% of genotype calls in polymorphic nucleotide positions contained missing data, they are spread through 13 SNPs out of 53 found in our dataset. Because the Isolation-with-Migration (IM) software used for parameter inferences does not consider any position containing missing data, we constructed a dataset in which missing data were removed (a reduced dataset). This reduction was done by withdrawing sequences of a specific locus when they contained most of the missing data of that locus, or withdrawing positions for which one or more individuals had no data. The procedure resulted in the loss of only one SNP. Table S1 presents the number of individuals and the coordinates of the alignment of the reduced dataset.

## **Admixture analyses**

We estimated admixture in 87 individuals from the Shimaa population using the Bayesian approach implemented in the Structure software (*21*), using a set of 106 Ancestry Informative Markers (AIMs, *23*) for 265 individuals from the following populations: Yoruba from Nigeria (n = 118), Euro-descendants from USA (n = 60) and Shimaas (n = 87). We performed three runs assuming three clusters (K = 3), using the correlated allele frequencies and admixture models, with 250,000 iterations after a burn-in of 50,000 in length; and lambda set to 1.0 and α parameters to be estimated for each of the three clusters.

## **1.3. Validation of the MCMC results**

We checked the convergence of our results based on three criteria: (1) We verified if the posterior distributions of parameters obtained with the three independent runs (using only different seeds) were similar; (2) The effective sample sizes (ESSs), a quantification of the number of independent observations made for each parameter, had to be all above 200; (3) The acceptance rate of proposed values along the Markov Chain had to be above 5%, which suggests that the parameter space was efficiently explored (*40*).

To test the possibility of reproducing the observed summary statistics (SuSt) under the estimated parameters of the MCMC model, we made a posterior predictive test (*27, 41*). We measured the discrepancy between the model and the data by a test quantity yielding a final Bayesian P-value, which is interpreted as the probability of accepting the null hypothesis that our data were generated by our model. To this end, we performed 10000 simulations with the parameter values random drawn from the posterior distributions. We then calculated the SuSt for each simulation to obtain their null distribution against which we tested the observed SuSt, obtaining a Bayesian P-value for each statistic. Finally, we combined the probabilities of the single statistics into a global P-value, using the method of Voight et al. (*42*) that considers the non-independence of the statistics. We used the fastsimcoal (*45*) and the arlsumstat (*44*) software within the ABCToolBox program (*46*) to simulate the IM model with 10 loci, with the same number of base pairs and chromosomes of our dataset, and to calculate the SuSt, respectively. The test was performed using an R script to run within ABCToolBox (developed by the Ferrara population genetics group participating in this study).

## **Inferences using Approximate Bayesian Computation (ABC)**

We used the program fastsimcoal within the ABCToolBox to simulate the IM model with 10 loci, with the same number of base pairs and chromosomes of our dataset, under the ABC (5x10^5^ simulations) and ABC_rec (7x10^5^ simulations) models. The values of the effective population sizes (N_e_), divergence time (t), and s parameters were drawn from uniform prior distributions set with similar range to the prior distribution used in the MCMC analyses. For the migration rates (m_i_) we used a loguniform distribution that is not available for the IM program, to have an equal coverage of the prior interval. With this resource available, we chose to use a wider prior distribution than we used with the IM program. For the mutation rate, we used a hyperprior distribution, that is, the mutation rates were sampled from a gamma distribution with α=12.46 and mean sampled from the uniform distribution specified in Table S2. The prior distribution for the recombination rate per bp per generation was set as a lognormal distribution with mean = 1.31x10^-8^ and standard deviation =1.78 (*42*). Prior distributions are given in Table S2.

We used the following SuSt for the ABC analyses: (i) the number of SNPs in each population; (ii) the mean intrapopulational pairwise differences (π, *47*); (iii) the Tajima´s D (*48*), which is an indicator of the proportion of rare and common alleles in the sample, being informative about the demographic history of the population, and ; (iv) the mean pairwise differences between populations (*49*); (v) the F_ST_ between populations (*50, 51*); and (vi) the proportion of mutations shared between populations (*52*). All these SuSt were calculate with arlsumstat inside ABCToolBox, except for the proportion of shared mutations, for which we developed Perl scripts to run within the ABCToolBox (available on www.ldgh.com.br). Table S3 presents the SuSt estimates for the observed data. We compared the posterior distributions obtained using four different sets of SuSt, as follows: (set i) all the SuSt for each locus, totaling 90 SuSt; (set ii) the averages and standard deviations of the SuSt calculated over the 10 loci, totaling 18 SuSt; (set iii) partial least-square (PLS, *53*) components extracted from set i; (set iv) PLS components extracted from set ii. The PLS is used to reduce the dimensionality of the summarized dataset and to break down the correlations among the different SuSt. The best number of PLS components is chosen such as the addition of more components do not reduce the root mean square error (RMSE) for the parameters predicted from these components (*53*). To generate the PLS components of set iii and set iv and transform the simulated and observed sets of statistics into PLS components we used ABCToolBox.

For parameter estimation, we calculated the Euclidian distance between the simulated and observed SuSt and retained the 1% of the total simulations corresponding to the shortest distances. Posterior probability for each parameter was estimated using a weighted local regression (*28*), after a logtan transformation to prevent the posterior distribution to exceed the limits of the prior distribution (*54*). These steps were done using a version of the makepd4 R script, freely available at http://www.rubic.rdg.ac.uk/~mab/stuff and modified by the Ferrara’s group.

## **Validation of ABC results**

We assessed the quality of the estimated parameters through several methods. First, we calculated the determination coefficient R² (the proportion of parameter variance explained by the summary statistics) computed through all simulations to determine whether the chosen SuSt contain enough information to estimate model parameters.

We also constructed Pseudo-Observed Datasets (PODS) to access the accuracy of the mode estimates of model parameters. To this end, we generated 1000 simulated datasets using the mode of posterior distributions as demographic parameters. Each of these datasets was considered as pseudo-observed data, which was analyzed with the total simulated datasets previously obtained for ABC estimation with the real observed data. Then, we computed the relative bias and relative mean square error (RMSE) that depend, respectively, on the sum of differences, and on the sum of squared differences, between the 1000 estimates of each parameter obtained from the pseudo-observed dataset, and the respective modes estimated from the observed data (‘true values’). A value of 0 means that the mode estimated the parameter with no bias, positive and negative values reflect, respectively, biases towards overestimation and underestimation. We also calculated the factor 2 statistic, defined as the proportion of the 1000 estimates that falls within the interval bounded by half and twice the true value; and the 90% coverage, defined as the proportion of times the true value is within the 90% credible interval of the 1000 estimates (*41, 55*). These statistics were performed by R scripts developed by the Ferrara population genetics group participating in this study.

Finally, we used the same posterior predictive test described in the “*Validation of the MCMC results*” section, to evaluate if the observed summary statistics could be reproduced under the estimated parameters of our ABC_rec and ABC models.

# **RESULTS**

## **2.1. Validation of MCMC results**

The convergence of MCMC simulations of the IM program were confirmed following the criteria specified in the Materials and Methods section: (1) We verified that the posterior distributions of parameters obtained with the three independent runs (using only different seeds) were similar (Figures 2-3); (2) The effective sample sizes (ESSs) were all above 1000 (Table S4); (3) The acceptance rates of proposed values obtained with the Markov Chain were all above 6% (Table S5).

From our posterior predictive test we obtained a global P-value of 0.45, which means that we could reproduce the observed data under the specific demographic scenario described by our model parameters.

The posteriors of the migration rates (m_1_ and m_2_) obtained with MCMC were flat, suggesting that our dataset does not contain sufficient information to infer migration rates.

However, the comparison between estimations assuming no gene flow (M = 0) and gene flow (M = 10) as priors were important to check if changes of the priors for the migration rate produce different effective sizes estimations (*39*). Figures 2 and 3 show that both MCMC estimations (without and with gene flow) yield essentially the same distribution for the estimated model parameters.

Additionally, we reduced the range of the priors to test if this would produce better curves for the parameters that did not yield good estimates, but this did not change any of the results.

## **2.2. Validation of ABC results and comparison between ABC and ABC_rec**

The estimates obtained from the four sets of SuSt (See Section 1.4) yielded similar posterior distributions. We chose to report the results obtained with 10 PLS components extracted from 90 summary statistics (set iii) for the ABC_rec and the total of 90 SuSt (set i) for the ABC based on the following criteria: (1) the posterior distribution curves obtained using the weighted local regression of Beaumont et al. (*28*) was similar to the posterior distribution curves obtained without using the regression; (2) the peaks of the posterior distribution curves were sharper than using the other sets of SuSt; (3) the estimates of posterior distributions retaining 0.5 and 1% of the total simulations yielded similar results.

By performing the posterior predictive test for ABC and ABC_rec models, we obtained a global P-value of 0.41 for both models, confirming that our inferred parameters can well reproduce the observed data.

Table S6 shows the determination coefficient R² (the proportion of parameter variance explained by the summary statistics) obtained for our estimated parameters. Even if these values are nominally low, they contain information for parameters estimation. Bertorelle et al. (*43*) have emphasized that suchlike the assignment of individuals to the correct population can be made even when a small fraction of variation is attributed to between-population differences (as in the case of the apportionment of human diversity), also small fractions of the explained variation (R²), are sufficient for reasonably correct parameter estimates, given enough data.

Table S7 presents the validation statistics calculated for each of the estimated demographic parameters from the simulated PODS, using the modes as true values for ABC (A) and ABC_rec (B). Except for the migration parameters, both relative bias and relative RMSE are low. Noteworthy, the positive relative biases for the time of divergence (t) between Quechuas and Shimaas suggest that this parameter is overestimated by our ABC analysis, which implies that the true divergence time is closer to the lower estimates obtained with the MCMC results (based on the whole dataset and not only on summary statistics as in the case of our ABC analyses). Also, with the exception of the migration parameters, the 90% coverage, calculated to assess the quality of the posterior distributions, was almost total for all the estimated parameters.

Our results from ABC_rec suggest that adding the recombination to the model yield very similar results for the divergence time and N_A_, but the ABC_rec yield less informative posterior densities for the s and N_2_ (Shimaa effective size) parameters (Figure 3). Figure S4 presents a comparison of SuSt obtained from simulations from the two models (ABC and ABC_rec). Overall, while medians of the SuSt are similar, SuSt obtained with ABC have higher variance than the ABC_rec.

As with the MCMC inference, we reduced the range of the priors to test if this would produce better curves for the parameters that did not yield good estimates, but this did not change any of the results.

As expected (*28*), the posterior distributions obtained with the ABC methodology have broader credible intervals than that obtained with the MCMC approach. Nonetheless, we can observe the same trends obtained with the likelihood-based MCMC approach, based on the entire dataset, and not only on summary statistics. Thus, ABC confirms the results obtained by the MCMC approach.

# **SUPPLEMENTARY TABLES**

Table S1. Re-sequenced regions for the reduced dataset (see text). Reported positions referred to the GenBank accession numbers.

| Loci | GenBank Accession Number Build 37.2 | Coordinates Region A^1^ | Coordinates Region B^1^ | Length of the segment² | N Quechuas^3^ | N Shimaas^4^ |
| --- | --- | --- | --- | --- | --- | --- |
| 1 | NT_029419.12 | 9914879 - 9915449 / 9915529 - 9916018 | 9924598- 9925514 | 1977 (155) | 11 | 10 |
| 2 | NT_007592.15 | 14702908 - 14703878 | 14711738 - 14711979 / 14712478 - 14712815 | 1551 (0) | 7 | 7 |
| 3 | NT_006576.16 | 9966053 - 9966153 / 9966214 - 9967019 | 9974876 - 9975580 | 1612 (285) | 8 | 9 |
| 4 | NT_011387.8 | 7613481 – 7614536 | 7604239 - 7605450 | 2268 (5) | 8 | 10 |
| 5 | NT_034772.6 | 36519803 - 36521221 | 36511314 - 36512431 | 2537 (74) | 11 | 10 |
| 6 | NT_007819.17 | X | 8324058 - 8325457 | 1400 (0) | 9 | 9 |
| 7 | NT_011109.16 | 3541640 – 3543067 | 3550482 - 3551903 | 2847 (0) | 10 | 10 |
| 8 | NT_010393.16 | 17909417 - 17910230 | 17900152 - 17901229 | 1891 (85) | 11 | 10 |
| 9 | NT_030059.13 | 70199409 – 70199930 / 70199969 - 70200228 | 70190335 - 70191212 | 1639 (59) | 10 | 9 |
| 10 | NT_016354.19 | 29311385 - 29311931 / 29312036 - 29312261 / 29312287 - 29312320 | 29321155 - 29321952 | 1605 (15) | 8 | 7 |

^1^ start – end positions of regions A and B. The symbol “/” means a segment with missing data that was removed, except for the region B of locus 2, that has two sequenced segments separated by an intervening segment.

^2^ In parenthesis are the number of base pairs removed due to missing data (represented by / in the coordinate regions).

^3^ Final number of Quechua individuals per locus after removal of missing data. The following were removed. Locus 2: QA38, QT135, QT140, QT1046; Locus 3: QT80, QT135, QT148; Locus 4: QT119, QT1046, QA38; Locus 6: QT148, QA38; Locus 7: QA38; Locus 9: QT140; Locus 10: QT140, QT160, QT1046.

^4^ Final number of Shimaa individuals per locus after removal of missing data. The following were removed. Locus 2: SH171, SH419, SH496; Locus 3: SH496; Locus 6: SH309; Locus 9: SH309; Locus 10: SH419, SH311, SH487.

Table S2. Ranges of prior distributions for the parameters used in MCMC and ABC methods.

| Demographic Parameters | | Prior range  MCMC^1^ ABC^2^ | |
| --- | --- | --- | --- |
| Time split (t) | [0 – 34014] | | [250 – 30000] |
| N_e_ Ancestral (N_A_) | [0 – 58107] | | [10 – 57500] |
| N_e_ Quechua (N_1_) | [0 – 58107] | | [10 – 57500] |
| N_e_ Shimaa (N_2_) | [0 – 40860] | | [10 – 40000] |
| Size split (s) | [0 – 1] | | [0.001 – 0.999] |
| Migration rates (m_1_, m_2_) | [0 – 0.00037] | | [10^-7^ – 10^-1^] ^a^ |
| Mean mutation rate (µ) | - | | [5x10^-9^ – 4.76x10^-8^]^b^ |
| Recombination rate (r)^c^ | - | | [1.31x10^-8^, 1.78]^d^ |
| Recombination rate intervening 1 (r_1_)^e^ | - | | [1.05x10^-4^, 1.78]^d^ |

Time split in years; Population size in number of individuals; Migration rates are per gene per generation; Mean mutation rate per site per generation; Recombination rate per site per generation; used only in the ABC_rec model.

^1^Prior ranges used for the MCMC approach converted to demographic quantities.

^2^Prior ranges used for the ABC and ABC_rec analyses.

^a^Loguniform distribution.

^b^Mean of the gamma distribution.

^c^Recombination rate within regions A and B.

^d^Mean and standard deviation of a lognormal distribution.

^e^Recombination rate of the intervening segment. We modeled the recombination rate of the intervening segment (~8kb) as if it had 1bp (to skip the time to simulate the intervening segment). Thus, the mean of the r_1_ lognormal distribution was 1.31x10^-8^ multiplied by 8000 bp. Locus 2 had one plus intervening segment (500bp). We used a lognormal distribution with mean 6.54x10^-6^ and standard deviation 1.78 as prior of r_2_ (the recombination rate for this second intervening segment), modeled as described above.

Table S3. Summary statistics for each locus calculated from the reduced dataset.

| Loci^a^ | Population | N | SNPs | Shared SNPs^b^ | | Tajima´s D | π | π_QT_SH_^c^ | F_ST_ |
| --- | --- | --- | --- | --- | --- | --- | --- | --- | --- |
| Locus 1 | Quechua | 11 | 4 | 1  1 | (0.25) | -1.667 | 0.446 | 0.350 | -0.021 |
|  | Shimaa | 10 | 1 |  |  | -0.086 | 0.268 |  |  |
| Locus 2 | Quechua | 7 | 5 | 4 | (0.8) | -0.389 | 1.396 | 1.112 | -0.047 |
|  | Shimaa | 7 | 4 | 4 |  | -0.848 | 0.934 |  |  |
| Locus 3 | Quechua | 8 | 2 | 2 | (1) | 1.085 | 0.858 | 0.812 | -0.030 |
|  | Shimaa | 9 | 2 | 2 |  | 0.988 | 0.817 |  |  |
| Locus 4 | Quechua | 8 | 7 | 5 | (0.71) | 0.054 | 2.142 | 1.500 | 0.101 |
|  | Shimaa | 10 | 5 | 5 |  | -1.780 | 0.589 |  |  |
| Locus 5 | Quechua | 11 | 10 | 7 | (0.7) | -0.429 | 2.398 | 1.886 | -0.003 |
|  | Shimaa | 10 | 7 | 7 |  | -0.981 | 1.384 |  |  |
| Locus 6 | Quechua | 9 | 2 | 2 | (1) | 1.894 | 1.033 | 1.000 | 0.036 |
|  | Shimaa | 9 | 2 | 2 |  | 1.318 | 0.895 |  |  |
| Locus 7 | Quechua | 10 | 8 | 0 | (0) | -1.105 | 1.516 | 0.800 | 0.052 |
|  | Shimaa | 10 | 0 | 0 |  | - | 0.000 |  |  |
| Locus 8 | Quechua | 11 | 6 | 3 | (0.5) | 0.659 | 1.996 | 1.563 | -0.007 |
|  | Shimaa | 10 | 3 | 3 |  | 0.970 | 1.153 |  |  |
| Locus 9 | Quechua | 10 | 2 | 2 | (1) | 1.639 | 0.958 | 0.966 | -0.036 |
|  | Shimaa | 9 | 2 | 2 |  | 1.949 | 1.046 |  |  |
| Locus 10 | Quechua | 8 | 6 | 6 | (1) | 1.704 | 2.708 | 2.607 | -0.050 |
|  | Shimaa | 7 | 6 | 6 |  | 1.679 | 2.769 |  |  |
| Average | Quechua | - | 52 | 32 | (0.696) | 0.344 | 1.545 | 1.259 | -0.0007 |
|  | Shimaa | - | 32 | 32 |  | 0.321 | 0.986 |  |  |

^a^More information about the loci sequenced can be found in Table S1 and in Scliar et al. 2012.

^b^In parenthesis is the proportion of shared SNPs between populations as in Patin et al. (*50*), defined as follows: $\sum_{ij}$= $\frac{sSij}{Si+Sj-sSij}$ , where *S_i_* is the number of segregating sites in population *i*, *S_j_* the number of segregating sites in population *j* and *_s_S_ij_* the number of segregating sites shared between populations *i* and *j*.

^c^The average number of pairwise differences between Quechuas (QT) and Shimaas (SH) sequences.

Table S4. Effective sample size (ESS) estimates for each parameter for the six independent runs of the MCMC analyses.

| Runs | L[P()]^a^ | q1^b^ | q2 | qA | T | s | m_1_ | m_2_ |
| --- | --- | --- | --- | --- | --- | --- | --- | --- |
| 1 (M=10) | 47328 | 1541985 | 335127 | 36496 | 12902 | 129108 | 319379 | 365691 |
| 2 (M=10) | 176016 | 309416 | 1340737 | 44305 | 4329 | 262293 | 354155 | 271951 |
| 3 (M=10) | 44299 | 285256 | 825127 | 38687 | 1412 | 33072 | 151396 | 45979 |
| 1 (M=0) | 130429 | 314786 | 252971 | 109768 | 1491 | 122105 | - | - |
| 2 (M=0) | 255330 | 280406 | 629902 | 25190 | 3950 | 36836 | - | - |
| 3 (M=0) | 607159 | 1007849 | 749970 | 32866 | 1262 | 294888 | - | - |

^a^ Log(P) = Log(P(Data|Genealogy)) + Log(P(Genealogy|Parameters)). This value provides a general idea of how well the Markov chain is mixing.

^b^ q corresponds to the θ parameter in the IM software nomenclature. Population 1 is the Quechua and 2 the Shimaa. Population A corresponds to the ancestral population.

Table S5. Update acceptance rates (%) for each parameter along the six independent runs of the MCMC analyses.

|  | q1^a^ | q2 | qA | t | s | m_1_ | m_2_ |
| --- | --- | --- | --- | --- | --- | --- | --- |
| 1 (M=10) | 82.16 | 74.36 | 6.32 | 6.58 | 53.4 | 92.42 | 92.72 |
| 2 (M=10) | 82.72 | 75.49 | 6.31 | 6.35 | 54.69 | 92.61 | 92.8 |
| 3 (M=10) | 82.18 | 74.75 | 6.30 | 6.66 | 53.66 | 92.5 | 92.69 |
| 1 (M=0) | 83.46 | 76.45 | 6.32 | 6.16 | 55.07 | - | - |
| 2 (M=0) | 83.22 | 76.11 | 6.32 | 6.28 | 54.38 | - | - |
| 3 (M=0) | 83.73 | 76.74 | 6.31 | 6.13 | 55.1 | - | - |

^a^ q corresponds to the θ parameter in the IM software nomenclature. Population 1 is the Quechua and 2 the Shimaa. Population A corresponds to the ancestral population.

Table S6. Determination coefficient R² obtained for our estimated parameters

| Demographic  Parameters | R² ABC | R² ABC_rec |
| --- | --- | --- |
| Time split (t) | 0.04 | 0.05 |
| N Ancestral (N_A_) | 0.53 | 0.52 |
| N Shimaa (N_2_) | 0.10 | 0.08 |
| S | 0.05 | 0.03 |
| N Quechua (N_1_) | 0.08 | 0.06 |
| Migration rate (m_1_) | 0.06 | 0.09 |
| Migration rate (m_2_) | 0.07 | 0.09 |

Table S7. Validation of ABC analyses using 1000 Pseudo Observed Datasets (PODS). PODS were obtained by simulations conditioning on the modes of the estimated posterior densities under the ABC model (without recombination, A) and the ABC_rec model (B).

**A.** Mode estimates (true values) under the ABC model

|  | Time Split | N_A_ ^a^ | N_1_ | N_2_ | s | m_1_ | m_2_ |
| --- | --- | --- | --- | --- | --- | --- | --- |
| True Value | 132 | 7657 | 99866 | 12898 | 0.89 | 0.0000001 | 0.0000001 |
| Bias | 1.051 | -0.749 | -0.647 | 0.709 | -0.404 | 8.225 | 11.663 |
| RMSE^b^ | 2.505 | 0.760 | 0.740 | 1.929 | 0.542 | 19.375 | 30.285 |
| 90% coverage | 0.965 | 0.941 | 0.973 | 1 | 0.943 | 0.256 | 0.19 |
| Factor2 | 0.634 | 0.041 | 0.219 | 0.57 | 0.568 | 0.072 | 0.079 |

**B.** Mode estimates (true values) ABC_rec model.

|  | Time Split | N_A_ | N_1_ | N_2_ | s | m_1_ | m_2_ |
| --- | --- | --- | --- | --- | --- | --- | --- |
| True Value | 135 | 8439 | 19330 | 21281 | 0.378 | 0.0000001 | 0.0000001 |
| Bias | 1.690 | -0.695 | 0.986 | 0.376 | 0.230 | 1.990 | 2.121 |
| RMSE^b^ | 3.295 | 0.705 | 2.109 | 1.186 | 0.903 | 4.527 | 4.850 |
| 90% coverage | 0.940 | 0.983 | 1 | 1 | 1 | 0.318 | 0.310 |
| Factor2 | 0.324 | 0.052 | 0.342 | 0.450 | 0.456 | 0.193 | 0.186 |

Population size in number of chromosomes and time split in generations. Effective sizes of: N_A_ - ancestral population, N_1_ – Quechua population, and N_2_ – Shimaa population. Migration rates: m_1_ and m_2_.

^b^ The bias and RMSE (Root Mean Square Error) are expressed in relative units.

1. **SUPPLEMENTARY FIGURES**


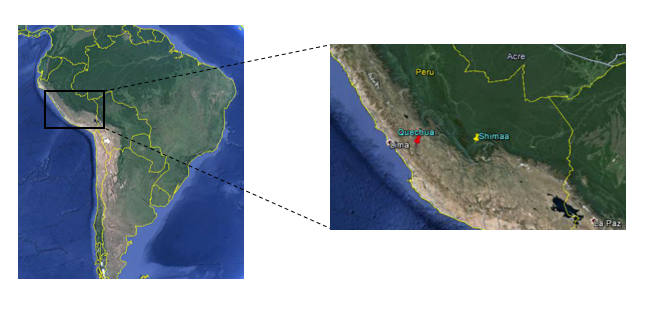


Figure S1. Location of the sampled populations Quechua (red pinpoint) and Shimaa (yellow pinpoint).


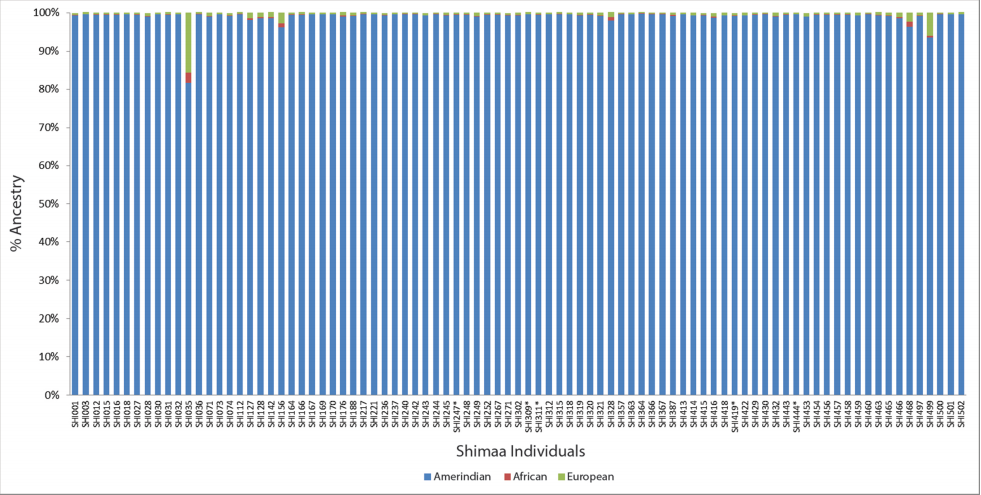


Figure S2. Percentage of Native American, African, and European ancestry for 87 individuals sampled from the Shimaa population. *Individuals re-sequenced in the present work.


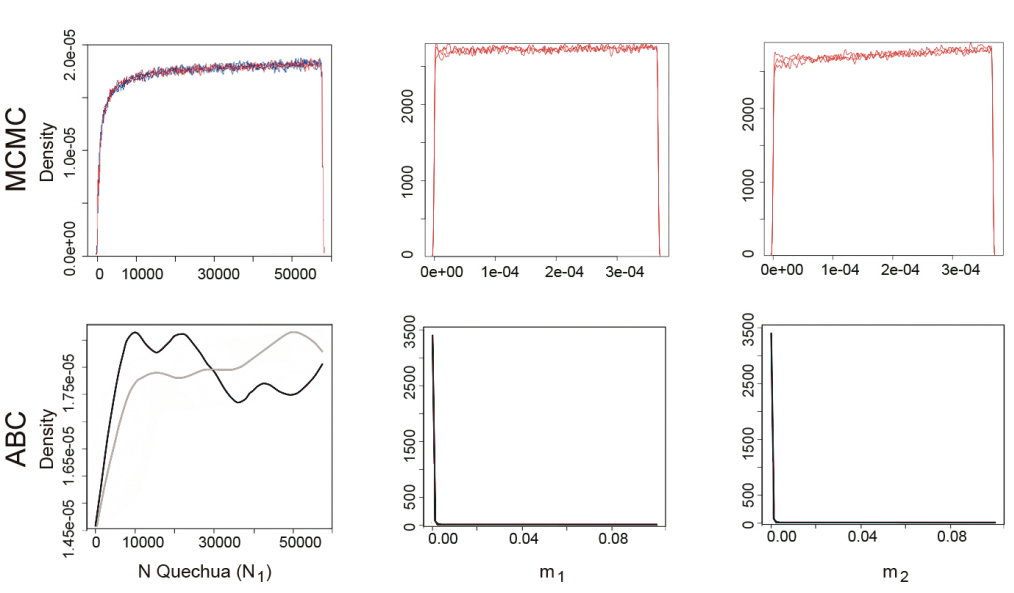


Figure S3. Posterior probability densities obtained by MCMC method and by the Approximate Bayesian Computation method (ABC) for the parameters N Quechua effective size (**N_1_**), migration rate from Quechua to Shimaa (**m_1_**), and migration rate from Shimaa to Quechua (**m_2_**). **N_1_** are in number of individuals. **MCMC plots**: Red: three independent runs with migration rate parameters M_i_=10; Blue: three independent runs with migration rate parameters M_i_=0. **ABC plots**: Gray: model without recombination (ABC); Black: model with recombination (ABC_rec).


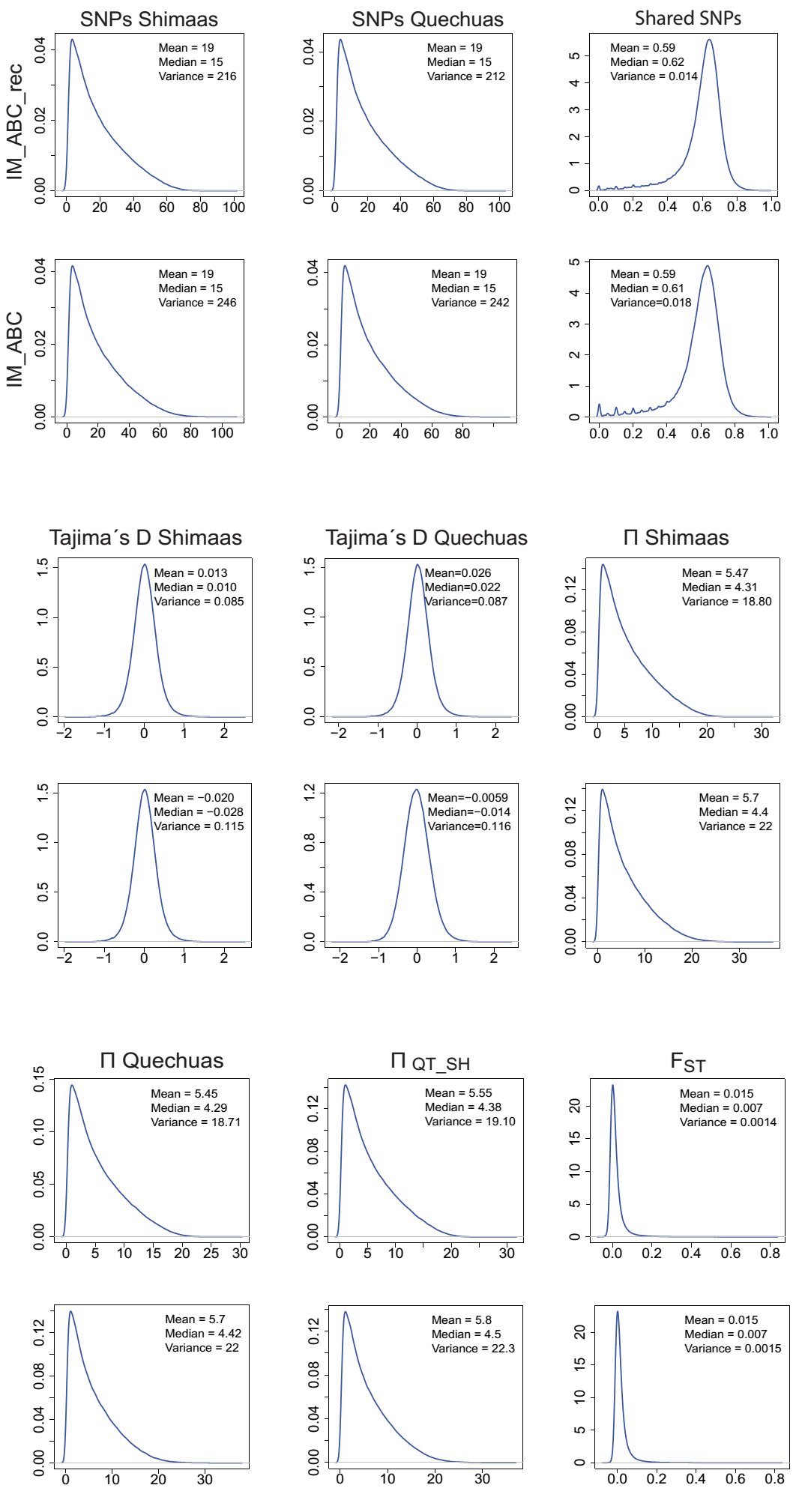


Figure S4. Comparison between the distributions of summary statistics obtained from simulations of the IM model by the ABC framework considering recombination (ABC_rec) and not considering recombination (ABC).

1. **REFERENCES**
2. Tajima F: **Evolutionary relationship of DNA sequences in finite populations.** *Genetics* 1983, **105**:437–60.
3. Tajima F: **Statistical method for testing the neutral mutation hypothesis by DNA polymorphism.** *Genetics* 1989, **123**:585–95.
4. Nei M, Li WH: **Mathematical model for studying genetic variation in terms of restriction endonucleases.** *Proc Natl Acad Sci U S A* 1979, **76**:5269–73.
5. Weir BS, Cockerham CC: **Estimating F-Statistics for the Analysis of Population Structure**. *Soc Study Evol Stableciety* 1984, **38**:1358–1370.
6. Michalakis Y, Excoffier L: **A generic estimation of population subdivision using distances between alleles with special reference for microsatellite loci.** *Genetics* 1996, **142**:1061–4.
7. Patin E, Laval G, Barreiro LB, Salas A, Semino O, Santachiara-Benerecetti S, Kidd KK, Kidd JR, Van der Veen L, Hombert J-M, Gessain A, Froment A, Bahuchet S, Heyer E, Quintana-Murci L: **Inferring the demographic history of African farmers and pygmy hunter-gatherers using a multilocus resequencing data set.** *PLoS Genet* 2009, **5**:e1000448.
8. Wegmann D, Leuenberger C, Excoffier L: **Efficient approximate Bayesian computation coupled with Markov chain Monte Carlo without likelihood.** *Genetics* 2009, **182**:1207–18.
9. Hamilton G, Currat M, Ray N, Heckel G, Beaumont M, Excoffier L: **Bayesian estimation of recent migration rates after a spatial expansion.** *Genetics* 2005, **170**:409–17.
10. Neuenschwander S, Largiadèr CR, Ray N, Currat M, Vonlanthen P, Excoffier L: **Colonization history of the Swiss Rhine basin by the bullhead (Cottus gobio): inference under a Bayesian spatially explicit framework.** *Mol Ecol* 2008, **17**:757–72.
