# Supplementary material for: Bayesian inferences suggest that Amazon Yunga Natives diverged from Andeans less than 5000 ybp: implications for South American prehistory
Source: BMC Evol Biol. 2014 Sep 30;14:174. doi: 10.1186/s12862-014-0174-3 (PMC4189748; doi:10.1186/s12862-014-0174-3)
Supplement: Supplementary file 2 — References used to prepare Figure 2 . [file 12862_2014_174_MOESM2_ESM.docx]

**Additional file 2**

**Title:** BAYESIAN INFERENCES SUGGEST THAT AMAZON YUNGA NATIVES DIVERGED FROM ANDEANS LESS THAN 5000 YBP: IMPLICATIONS FOR SOUTH AMERICAN PREHISTORY

Marilia O Scliar^1^, Mateus H Gouveia^1^, Andrea Benazzo^2^, Silvia Ghirotto^2^, Nelson J Fagundes^3^, Thiago P Leal^1^, Wagner CS Magalhães^1^, Latife Pereira^1^, Maira R Rodrigues^1^, Giordano Soares-Souza^1^, Lilia Cabrera^4^, Douglas E Berg^5^, Robert H Gilman^4,6,7^, Giorgio Bertorelle^2^, Eduardo Tarazona-Santos^1^

^1^ Departamento de Biologia Geral. Instituto de Ciências Biológicas, Universidade Federal de Minas Gerais. Brazil.

^2^ Dipartimento di Scienze della Vita e Biotecnologie. Università di Ferrara. Italy.

^3^ Departamento de Genética. Instituto de Biociências. Universidade Federal do Rio Grande do Sul. Brazil.

^4^ Asociación Benéfica PRISMA. Lima. Peru.

^5^ Department of Molecular Microbiology, Washington University School of Medicine, St. Louis, Missouri and Department of Medicine, University of California San Diego, California, USA.

^6^ Bloomberg School of Public Health, Johns Hopkins University. Baltimore. Maryland. USA.

^7^ Universidade Peruana Cayetano Heredia. Lima. Peru.

**REFERENCES FOR HISTORICAL EVENTS PRESENTED IN FIGURE 2**

Aldenderfer MS: **High elevation foraging societies**. In *The Handbook of South American Archaeology.* Edited by Silverman H, Isbell WH. New York: Springer; 2008: 131-143.

Beresford-Jones DG, Heggarty P: **Broadening our horizons: towards an interdisciplinary prehistory of the Andes**. In *Archaeology and Language in the Andes*. Edited by Heggarty P, Beresford-Jones DG. Oxford: Oxford University Press; 2012: 57–84.

Bruhns KO: *Ancient South America*. Cambridge: Cambridge University Press; 1994.

Burger RL: **Chavín de Huántar and its sphere of influence**. In *The Handbook of South American Archaeology.* Edited by Silverman H, Isbell WH. New York: Springer; 2008: 681-703.

Butters LJC, Castillo SU: **The Mochicas**. In *The Handbook of South American Archaeology.* Edited by Silverman H, Isbell WH. New York: Springer; 2008: 707-729.

Church WB, von Hagen A: **Chachapoyas: cultural development at an Andean cloud forest crossroads**. In *The Handbook of South American Archaeology.* Edited by Silverman H, Isbell WH. New York: Springer; 2008: 903-926.

Covey RA: **The Inca Empire**. In *The Handbook of South American Archaeology.* Edited by Silverman H, Isbell WH. New York: Springer; 2008: 809-830.

D´Altroy TN, Schreiber K: **Andean Empires**. In *Andean Archaeology*. Edited by Silverman H. Oxford: Blackwell Publishing; 2004: 255-279.

Deboer WR: **Ceramic assemblage variability in the Formative of Ecuador and Peru**. In *Archaeology of Formative Ecuador*. Edited by Quilter J, Burger RL, Raymond JS. Washington: Dumbarton Oaks Research Library and Collection; 2003: 289-336.

Dillehay TD, Bonavia D, Kaulicke P: **The first settlers**. In *Andean Archaeology*. Edited by Silverman H. Oxford: Blackwell Publishing; 2004: 16-34.

Finucane BC: **Maize and sociopolitical complexity in the Ayacucho Valley, Peru**. *Curr Anthropol* 2009, **50**:535–545.

Grobman A, Bonavia D, Dillehay TD, Piperno DR, Iriarte J, Holst I: **Preceramic maize from Paredones and Huaca Prieta, Peru.** *Proc Natl Acad Sci U S A* 2012, **109**:1755–9.

Haas J, Creamer W: **Cultural transformations in the Central Andean Late Archaic**. In *Andean Archaeology*. Edited by Silverman H. Oxford: Blackwell Publishing; 2004: 35-50.

Haas J, Creamer W, Huamán Mesía L, Goldstein D, Reinhard K, Rodríguez CV: **Evidence for maize (Zea mays) in the Late Archaic (3000-1800 B.C.) in the Norte Chico region of Peru.** *Proc Natl Acad Sci U S A* 2013, **110**:4945–9.

Hastorf CA: **The Formative Period in the Titicaca Basin**. In *The Handbook of South American Archaeology.* Edited by Silverman H, Isbell WH. New York: Springer; 2008: 545-561.

Moore JD, Mackey CJ: **The Chimú Empire**. In *The Handbook of South American Archaeology.* Edited by Silverman H, Isbell WH. New York: Springer; 2008: 783-807.

Moseley ME: *The Incas and Their Ancestors; The Archaeology of Peru*. London: Thames & Hudson; 2001.

Oliver JR: **The archaeology of agriculture in ancient Amazonia**. In *The Handbook of South American Archaeology.* Edited by Silverman H, Isbell WH. New York: Springer; 2008: 185-216.

Pearsall DM: **Plant domestication and the shift to agriculture in the Andes**. In *The Handbook of South American Archaeology.* Edited by Silverman H, Isbell WH. New York: Springer; 2008: 105-120.

Perry L, Dickau R, Zarrillo S, Holst I, Pearsall DM, Piperno DR, Berman MJ, Cooke RG, Rademaker K, Ranere AJ, Raymond JS, Sandweiss DH, Scaramelli F, Tarble K, Zeidler JA: **Starch fossils and the domestication and dispersal of chili peppers (Capsicum spp. L.) in the Americas.** *Science* 2007, **315**:986–8.

Pickersgill B: **Domestication of plants in the Americas: insights from Mendelian and molecular genetics.** *Ann Bot* 2007, **100**:925–40.

Pineda RF: **The Late Preceramic and Initial Period**. In *Peruvian Prehistory: An Overview of Pre-Inca and Inca Society*. Edited by Keatinge RW. Cambridge: Cambridge University Press; 1988: 67-98.

Piperno DR: **The origins of plant cultivation and domestication in the New World tropics**. *Curr Anthropol* 2011, **52**:S453–S470.

Pozorski S, Pozorski T: **Early cultural complexity on the Coast of Peru**. In *The Handbook of South American Archaeology.* Edited by Silverman H, Isbell WH. New York: Springer; 2008: 607-631.

Pozorski T, Pozorski S: **Architecture and chronology at the site of Sechin Alto, Casma Valley, Peru**. *J F Archaeol* 2005, **30**:143–161.

Proulx DA: **Paracas and Nasca: regional cultures on the South Coast of Peru**. In *The Handbook of South American Archaeology.* Edited by Silverman H, Isbell WH. New York: Springer; 2008: 563-585.

Roosevelt AC, Housley RA, Silveira MI, Maranca S, Johnson R: **Eighth millennium pottery from a prehistoric shell midden in the brazilian Amazon.** *Science* 1991, **254**:1621–4.

Roosevelt AC, Lima da Costa M, Lopes Machado C, Michab M, Mercier N, Valladas H, Feathers J, Barnett W, Imazio da Silveira M, Henderson A, Silva J, Chernoff B, Reese DS, Holman JÁ, Toth N, Schick K: **Paleoindian cave dwellers in the Amazon: the peopling of the Americas**. *Science* 1996, **272**:373-384.

Shimada I: **Evolution of Andean diversity: regional formations (500B.C.E.-C.E.600)**. In *The Cambridge History of the Native Peoples of the Americas, Volume 3.* Edited by Salomon F, Schwartz SB. Cambridge: Cambridge University Press; 1999: 350-517.

Solis RS, Haas J, Creamer W: **Dating Caral, a preceramic site in the Supe Valley on the central coast of Peru.** *Science* 2001, **292**:723–6.

Stahl PW: **Animal domestication in South America**. In *The Handbook of South American Archaeology.* Edited by Silverman H, Isbell WH. New York: Springer; 2008: 121-130.

Stanish C: *Ancient Titicaca: The Evolution of Complex Society in Southern Peru and Northern Bolivia*. Berkeley: University of California Press; 2003.

Tykot R, Burger RL, van der Merwe NJ: **The importance of maize in Initial Period and Early Horizon Peru**. In *Histories of Maize*. Edited by Staller JE, Tykot R, Benz BF. Walnut Creek: Left Coast Press; 2006: 187-198.

Walker RS, Ribeiro LA: **Bayesian phylogeography of the Arawak expansion in lowland South America.** *Proc Biol Sci* 2011, **278**:2562–7.

Zeidler JA: **The Ecuadorian Formative.** In *The Handbook of South American Archaeology.* Edited by Silverman H, Isbell WH. New York: Springer; 2008: 459-488.

Zucchi A: **The Arawakan matrix: ethos, language, and history in native South America**. In *Comparative Arawakan Histories: Rethinking Language and Culture Area in Amazonia*. Edited by Hill JD, Santos-Granero F. Urbana: University of Illinois Press; 2002: 199-222.
